# What do we think we are doing: principles of coupled self-regulation in human-robot interaction (HRI)

**DOI:** 10.3389/fpsyg.2015.00929

**Published:** 2015-07-08

**Authors:** Idit Shalev, Tal Oron-Gilad

**Affiliations:** ^1^Department of Education and the Zlotowski Center of Neuroscience, Ben-Gurion University of the NegevBeer-Sheva, Israel; ^2^Department of Industrial Engineering and Management, Ben-Gurion University of the NegevBeer-Sheva, Israel

**Keywords:** human-robot interaction (HRI), embodied cognition, self-regulation, goal pursuit, teamwork

The use of domestic service robots is becoming widespread. While in industrial settings robots are often used for specified tasks, the challenge in the case of robots put to domestic use is to afford human-robot collaboration in a variety of non-predefined and different daily tasks. Herein, we aim at identifying and understanding the conditions that will facilitate flexible collaboration between humans and robots. Past research of social and personality psychology was mainly focused on individual's self-regulation, defined as the ability to govern, or direct attention, resources, or action toward the realization of a particular goal (Higgins, 1989; Kruglanski et al., 2002). There is evidence that pursuing goals with the presence of others influences self-control (Fishbach and Trope, 2005), however only little is known on dyadic processes of self-regulation. Additionally, whereas research of goal pursuit in social psychology has mainly been associated with general processes of the structure and function of goals (Gollwitzer and Bargh, 1996; Carver and Scheier, 1998; Kruglanski et al., 2002; Fishbach and Ferguson, 2007; Custers and Aarts, 2010), human-robot interaction involves pragmatic interpersonal dilemmas such as how to coordinate human-robot activity and what knowledge should be shared between humans and robots over the course of action. To fill this gap, in what follows, we will define the unique characteristics of what we term as *human-robot coupled self-regulation*, which has the unique features of a dyadic asymmetric team aimed to increase the affordances of an individual in different activities. We will describe the unique characteristics of human-robot interaction and its special challenges toward goal pursuit.

## Human and robot are a dyadic instrumental asymmetric team

Our first assumption is that self-regulation of a human-robot couple could be conceptualized as a unique team configuration. A team is “a distinguishable set of two or more people who interact, dynamically, interdependently, and adaptively toward a common and valued goal/objective/mission, who have each been assigned specific roles or functions to perform, and who have a limited life-span of membership” (Salas et al., 1992, p. 4; Salas et al., 2010). Team members have differentiated responsibilities and roles (Cannon-Bowers et al., 1993). Therefore, essential for a team's successful performance is the understanding of the abilities and behaviors of its members that fit their experience and unique expertise for the task at hand.

Because humans and robots differ in their level of agency (the capacity to act and do) and their level of experience (the capacity to feel and sense), (Gray and Wegner, 2012), we argue that their contribution to the team is not symmetric. Based on the reasoning that genuine authorship of an action or situation may not always be clear (Dijksterhuis et al., 2008), we suggest that defined requirements of person, robot, and situation are essential to reduce the expectation gap.

Our perspective is that human-robot collaboration should be viewed in terms of functionality, to extend possibilities for the kinds of goals that humans want to pursue. These instrumental relations between a person and her tool, used to increase the fit between person and environment, are termed affordances (Gibson, 1979). Following this view, we argue that robots can be perceived as self-regulatory tools to increase affordances across different situations (Koole and Veenstra, 2015). Our instrumental relational approach enables flexibility in tuning the robot's level of responsiveness and dominance in human-robot social contexts. For example, whereas the human member of the team holds a fixed ownership position, the robot's level of dominance could vary by user demands, or depending on the situation. To understand the usefulness of this principle, let us take for example 80 year old Mrs. Brown. She is physically fragile, but it is important for her to maintain an independent life style. This is why she has “Rupert,” a multi-functional platform robot that serves as her aid. When she leaves the house she may want “Rupert” to lead and find the safest walking path to the store, thus she may set it to high dominance and responsiveness, in case she startles. At home, she may not desire high level of proactive care-taking and leave “Rupert” to be on call.

## Concrete level of human-robot negotiation

Our second assumption is that human-robot coupled self-regulation is based on concrete rather than abstract level of agreement. Carrying out human-robot joint actions demands continuous coordination on at least five elements: (1) who takes part; (2) what is the role of each member; (3) what is the joint goal; (4) how does each team member contribute to the timing and synchronization; and (5) where the actions take place (Clark, 2005). To address this, the robot should identify where the focus of attention of the human is, to what degree the attention of the human is focused on team actions, and how to convey feedback. Similarly, the human needs to calibrate expectations from the robot, i.e., be invested in the robot's immediate action or approval of action, and how to respond to the robot's requests (Alami et al., 2005).

Coupled self-regulation of goals requires agreement on goal setting and goal striving as two basic phases in goal pursuit (Gollwitzer and Oettingen, 2011). Whereas, robots may act automatically from initiation to completion of the task, humans' possible reflection on their performance may involve conscious awareness and create new representations of behavior, thus leading to communication gaps (Baumeister and Bargh, 2014). According to the action identification theory, a specific action can be verbally identified and interpreted from different levels of abstraction, ranging from low-level identities that specify how the action is performed, to high-level identities that signify why the action is performed. For instance, a person who “drinks water” can identify it as “holding a glass” (low level), or as “relieving thirst” (high level) (Vallacher and Wegner, 1987, 1989). This helps explain why different action identifications by human and robot may lead to dissimilar systems of goals and means of attainment (Kruglanski et al., 2002; Shah et al., 2002).

To address these challenges, we suggest the use of multiple human-robot forms of communication to pursue the joint goal. Lohan et al. (2014) proposed a distinction between two kinds of actions: path-oriented and manner-oriented, that can be communicated via two different linguistic utterance styles. Whereas, in path-oriented utterances the goal is stressed, in manner-oriented utterances, the means of motion are emphasized (e.g., Talmy, 1991). In our example, Mrs. Brown and “Rupert” carry a recliner to the porch (Path-“let's move the chair to the porch” or Manner-“I want to read my book on the porch”). Suddenly the phone rings and Mrs. Brown wants to go and answer ((Path-“let me go get the phone” or Manner-“I need to answer this call”). “Rupert” must understand that the goal has changed and pause.

## Continuous and various communication forms over goal pursuit

Research indicates that professional and social interactions between team members can develop the team's social cognition (Klimoski and Mohammed, 1994). There is evidence that a team's fluent on-going communication regarding goal pursuit reduces the need for preexisting knowledge (Kozlowski and Bell, 2003). In social HRI, it is critical to generate many levels of interaction with the automation. Hence, the robot should always be present and aim to facilitate the goal, even if only to provide recommendations. In civil aviation, for example, communication is key especially if things turn out unexpectedly. In the Northwest 2009 incident in Minneapolis the automation had the capability, but was not designed to point out that the task was not performed as planned and that the pilots missed their destination. To borrow from our previous example, let us suppose Mrs. Brown wants to grab a pillow from the upper cabinet. The robot may not be able to reach so high, but it should continue to collaborate by providing feedback and advice; I cannot reach the uppermost cupboard (failure to complete task) but it is too dangerous for you to try to reach it on your own, if not urgent, perhaps we should call your son, or is there another pillow on a lower shelf?

Much of human communication over goal pursuit is based on social cues (e.g., gestures, and mimicry) that automatically generate social judgment and behavior (Chartrand and Bargh, 1999; van Baaren et al., 2003; Leander et al., 2010). Similarly, translation of social cues to social signals leads to inference of human intentions by robotic agents (Fiore et al., 2013). The relevance of automatic embodied cues for joint goal pursuit was demonstrated in human-human and human-robot synchronicity, suggesting that physical synchronicity is associated with experience of responsiveness and empathy (Sebanz and Knoblich, 2009; Cohen et al., 2010; Paladino et al., 2010; Boucher et al., 2012; Hoffman et al., 2014). Embodied communication is not only “used” by robots, but integrated in them to support both the recognition of the human's behavior and the generation of their behavior. Research of social signal processing and modeling multimodal communication, suggests that social and behavioral cues may be detectable from a machine, hence perceivable. Likewise, models of behavior are integrated in a way that a robot exhibits a more natural behavior, aiming at a more successful interaction with the human (Pentland, 2007; Vinciarelli et al., 2012).

However, despite emerging findings from the field of embodied cognition on the potential of physical and social cues as an alternative route for communication, it was also claimed that embodied cognition cues can lead to different patterns of activation across different contexts (Loersch and Payne, 2011), thus prediction of behavior may be difficult (Shalev, 2015). A possible way to address this limitation is to use robots in fixed context, where interpretation to human's embodied signals is less ambiguous. For example Loth et al. (2013), have demonstrated that bar staff responded to a set of two non-verbal signals. Foster (2014), indicated that robotic sensors can similarly detect and respond to these signals.

## Addressing the human-robot communication gap over goal pursuit

Individuals frequently use embodied cues for functional self-regulatory purposes (Balcetis and Cole, 2009; Schnall et al., 2010; Bargh and Shalev, 2012; Shalev, 2014). However, using embodied cues as diagnostic inputs (Williams et al., 2009; Ackerman et al., 2010; Meier et al., 2012; Robinson and Fetterman, 2015; Winkielman et al., 2015) may lead to human-robot miscommunications. For example, human speakers expect co-located listeners to link visually perceivable objects and the verbally described references to them. Thus, humans may expect a co-located robot to have the same visual-verbal linking abilities (e.g., look at the green object on the right), thus developers must integrate the robot's visual system with natural language components to enable this flow of communication (Kopp, 2010; Cantrell et al., 2012; Vollmer et al., 2013).

Furthermore, there is also anecdotal evidence of human-human communication misunderstandings in complex scenes. For example orientation can be relative to egocentric, or exocentric (absolute or relative) locations. Soldiers for example, are taught to communicate via the exocentric coordinates of the compass rose. However, most humans tend to naturally orient relative to their egocentric perspective, which may be difficult for robots to depict. Interestingly, Cassenti et al. (2012) found that instructors used exocentric references to direct the robot and that it improved their performance relative to egocentric-only commands.

To address this communication gap, we argue that shared database, sensors and multiple types of displays and interaction means (e.g., physiological measures, eye tracking, voice, touch, text, button presses etc.) can enrich the robot's capacity of perception and expression. Similarly, to reduce expectation issues, technology can shape the way the user acts on the robot, how individuals understand what to expect from it, and how they can interact with a robot to refine their mutual understanding of the task at hand. Providing the relevant information about the current state of the robot, the progress of the task, and of the surrounding environment, can facilitate successful performance. Similarly, education efforts need to convey the ambiguity of ongoing human-robot communication, particularly the robot's physical and data-driven limitations, and to encourage problem solving and novelty seeking.

### Conflict of interest statement

The authors declare that the research was conducted in the absence of any commercial or financial relationships that could be construed as a potential conflict of interest.
